# Exploring differences in the use of the statin choice decision aid and diabetes medication choice decision aid in primary care

**DOI:** 10.1186/s12911-017-0514-5

**Published:** 2017-08-10

**Authors:** Aimee Yu Ballard, Maya Kessler, Marianne Scheitel, Victor M. Montori, Rajeev Chaudhry

**Affiliations:** 1 Department of Medicine, Division of Primary Care Internal Medicine, Mayo Clinic, Rochester, MN USA; 2 Knowledge Delivery Center, Mayo Clinic, 200 First Street SW, Rochester, MN 55905 USA; 3 Knowledge and Evaluation Research Unit, Mayo Clinic, Rochester, MN USA; 4 Department of Medicine, Division of Endocrinology, Mayo Clinic, Rochester, MN USA

## Abstract

**Background:**

Shared decision making is essential to patient centered care, but can be difficult for busy clinicians to implement into practice. Tools have been developed to aid in shared decision making and embedded in electronic medical records (EMRs) to facilitate use. This study was undertaken to explore the patterns of use and barriers and facilitators to use of two decision aids, the Statin Choice Decision Aid (SCDA) and the Diabetes Medication Choice Decision Aid (DMCDA), in primary care practices where the decision aids are embedded in the EMR.

**Methods:**

A survey exploring factors that influenced use of each decision aid was sent to eligible primary care clinicians affiliated with the Mayo Clinic in Rochester, MN. Survey data was collected and clinician use of each decision aid via links from the EMR was tracked.

**Results:**

The survey response rate was 40% (105/262). Log file data indicated 51% of clinicians used the SCDA and 9% of clinicians used the DMCDA. Reasons for lack of use included lack of knowledge of the EMR link, not finding the decision aids helpful, and time constraints. Survey responses indicated that use of the tool as intended was low, with many clinicians only discussing decision aid topics that they found relevant.

**Conclusion:**

Although guidelines for both the treatment of blood cholesterol with a statin and for the treatment of hyperglycemia in type 2 diabetes recommend shared decision making, tools that facilitate shared decision making are not routinely used even when embedded in the EMR. Even when decision aids are used, their use may not reflect patient centered care.

**Electronic supplementary material:**

The online version of this article (doi:10.1186/s12911-017-0514-5) contains supplementary material, which is available to authorized users.

## Background

Shared decision making involves three essential elements: (1) awareness that a decision is required; (2) sharing of evidence about the pros and cons of different treatment options; and (3) a discussion of patient values and preferences [1]. Shared decision making is critical to patient-centered care [2], but implementing shared decision-making is not easy [3]. For this reason, decision aids, which do not guarantee shared decision-making, but have been shown to increase the likelihood clinicians engage patients in treatment decisions, have been developed [4]. Decisions aids can take many forms, but often involve a graphic presentation of the evidence of risks and benefits, and are used during a clinical encounter to facilitate discussion between clinicians and patients. In some practices electronic versions of decision aids have been incorporated into the electronic medical record (EMR) to facilitate use. However, even when embedded in the work-flow, decision aids are not routinely used [5]. Given the increasing emphasis on shared decision-making by organizations such as the National Quality Forum and the likely development of quality metrics based on the use of decision aids under the Affordable Care Act [6], understanding the factors that promote and impede use of decision aids in clinical practice is paramount. This study investigated patterns of use as well as barriers and facilitators of use for two decision aids: the Statin Choice Decision Aid (SCDA) and the Diabetes Medication Choice Decision Aid (DMCDA) in primary care practices affiliated with the Mayo Clinic.

## Methods

### Study design

We administered a survey to investigate factors that encouraged or discouraged use of the SCDA and the DMCDA by primary care clinicians at the Mayo Clinic, an academic tertiary healthcare center. Decision aid use was measured by querying log-file data that recorded the use of links to the decision aids from the EMR. The Mayo Clinic Institutional Review Board approved all study procedures.

### The statin choice decision aid and the diabetes medication choice decision aid

The SCDA and DMCDA were developed at the Mayo Clinic to encourage patient involvement in treatment decisions. In randomized trials, both decision aids have shown to increase patient knowledge and engagement and to decrease decisional conflict [7–11]. Both decision aids were designed with input from primary care clinicians practicing at the Mayo Clinic [12, 13].

Links to the decision aids are located in the EMR, in a section routinely used by primary care clinicians at the Mayo Clinic to ensure preventative services and tests for chronic conditions are up to date. Clinicians in primary care were notified by e-mail of the presence of the links to the decision aids when the links were initially added to the EMR, but no training on the use of the decision aids to facilitate shared decision-making was given.

#### The statin choice decision aid

The SCDA is a tool that helps clinicians and patients discuss the pros and cons of statin use. It graphically displays information about a patient’s estimated 10-year cardiovascular risk, the degree of risk reduction with a statin, and the likelihood of adverse events (https://statindecisionaid.mayoclinic.org). At the Mayo Clinic, each patient’s data from the EMR is imported into the tool in order to individualize presentation.

#### The diabetes medication choice decision aid

The DMCDA uses electronic issue cards to display the impact of different diabetes medications on daily routine, blood sugar control, risk of hypoglycemia, weight change, and cost (https://diabetesdecisionaid.mayoclinic.org). Patients and clinicians identify the best medication for a particular patient based on reviewing 2–3 of issue cards.

### Study participants

All primary care clinicians (nurse practitioners, physician assistants, physicians, and physicians in training) practicing in the divisions of Family Medicine or Primary Care Internal Medicine at Mayo Clinic in Rochester, Minnesota were eligible for participation in the study.

### Survey data

A 14-question survey on decision aid use was designed by the study team. Survey questions covered the following topics: use of the decision aid, barriers and facilitators to use, and type of use. The survey was administered electronically by Qualtrics and sent to all eligible primary care clinicians (262) via email on June 16th, 2015. Reminder emails (3) were sent, until the survey closed on September 16, 2015.

All survey questions were in a check box format. One survey question, in addition to the check box format, allowed additional data entry in a free-text format. Clinicians had to answer each question before advancing to the next question. If a clinician indicated that they had not used a decision aid, then they were not asked the specific questions pertaining to that decision aid. At the end of the survey, clinicians had the option to submit their answers. Only submitted surveys were available for analysis.

For some questions a Likert scale was used, with similar responses combined for the purpose of data analysis. Also for the purpose of analysis, survey responses detailing barriers and facilitators to use were subdivided into three categories—knowledge barriers and facilitators, attitudes barriers and facilitators, and external barriers and facilitators – based on the classification scheme used by Legare et al. Please see Additional file 1 for survey questions and detailed explanation of coding.

### Log file data

Data on decision aid use was captured for all clinicians by log file data that recorded use of the links to each decision aids from the EMR. The total number of uses as well as the number of distinct clinicians using each decision aid each month was captured for a 12 month period of time, from December 16th, 2014 to December 15th, 2015. This time period was chosen to capture the 6 months before the survey was first sent and the 6 months after the survey was first sent.

### Demographic data

Clinician characteristics including age, gender, and type of training (nurse practitioners, physician assistants, physicians, and physicians in training), and years in practice was obtained from administrative sources.

### Statistical analysis

Summary statistics were created using frequencies for categorical data and means and standard deviation for continuous data.

## Results

The response rate to the survey was 40% (105 out of 262). The majority of the respondents were physicians or physicians in training (82%). There were equal numbers of male and female respondents (50% male). The mean age of respondents was 41 years and respondents had an average of 14 years in practice. Demographic characteristics of survey respondents were similar to the demographic characteristics of the practice as a whole (see Table 1).

**Table 1:** Characteristics of Clinicians

Variable	Description	Survey Respondents *n* = 105% or m(SD)	All Clinicians *n* = 262% or m(SD)
Type of clinician	Nurse Practitioner	14.4%	16.0%
	Physician Assistant	3.8%	3.1%
	Physician	37.5%	35.1%
	Physician in Training	44.2%	45.8%
Sex	Male	49.5%	45.2%
Age	–	40.7 (12.2)	40.6 (12.0)
Years in Practice	–	13.6 (12.3)	13.5 (12.0)

**Fig. 1:**
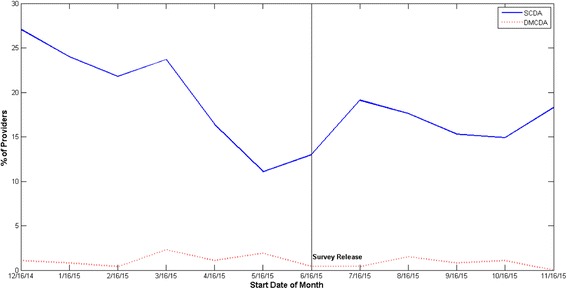
Percent of Survey Respondents who accessed DMCDA and SCDA per month

More clinicians used the SCDA than the DMCDA according to both the survey data (81% vs. 29%) and log file data (51% vs 9%). Log-file data indicated survey respondent’s use of the decision aids was slightly higher than use of the decision aids by the practice as a whole (see Table 2). There was no increase in use of either the SCDA or DMCDA by survey respondents after the survey was administered (see Fig. 1).

**Table 2:** Decision Aid Use, Log File Data

Time period	Survey Respondents (*n* = 105)				All Clinicians (*n* = 262)			
	DMCDA access		SCDA access		DMCDA access		SCDA access	
	# of times	% of clinicians	# of times	% of clinicians	# of times	% of clinicians	# of times	% of clinicians
Six months pre- survey	14	10.5%	552	55.2%	21	6.9%	1151	45.4%
Six months post-survey	11	7.6%	363	46.7%	12	3.4%	819	34.0%
Entire 12 months period	25	15.2%	915	62.3%	33	9.1%	1970	51.1%

Survey responses indicated that clinician lack of knowledge about the decision aids was a barrier to use, especially for the DMCDA. Clinicians were much less familiar with the DMCDA than with the SCDA (7% vs. 42%). Lack of awareness of the EMR link may have also contributed to infrequent use, although this was roughly equal for both the SCDA and DMCDA (47 vs. 43%). Attitudes that inhibited decision aid use included feeling the decision aid was not accurate and not helpful, but a minority of clinicians had these attitudes (see Table 3). Environmental factors also seemed to affect use of both decision aids, with time constraints being a barrier to use for both the DMCDA and SCDA (11% vs 21%).

**Table 3:** Decision Aid Use, Barriers and Facilitators of Use, and Type of Use, Survey Data (*n* = 105)

Domain	Category	DMCDA%	SCDA %
Use	Routinely used	28.4%	80.6%
Knowledge Barriers	Unfamiliar with decision aid	42.3%	6.9%
	Unaware of EMR link	43.1%	47.4%
Attitude Barriers	Not helpful	19.6%	15.8%
	Not accurate	1.0%	5.3%
Attitude Facilitators	Very useful	29.6%	56.4%
External Barrier	Time constraints	10.7%	21.1%
External Facilitator	Appropriate amount of information	85.2%	94.8%
	Often impacts treatment decision	22.2%	42.3%
Type of use	Only discuss topics patient is interested in	42.3%	17.9%
	Only discuss topics I find relevant	46.2%	60.3%
	Discuss all topics (use as intended)	11.5%	21.8%

Table 4 lists barriers noted by clinicians, recorded as free-text comments in the survey. For the SCDA barriers included forgetting to use it and feeling like it discouraged statin use in the face of guidelines that promote statin use. For the DMCDA barriers included problems with the format of the decision aid, lack of experience with some of the medications, and lack of applicability to a clinician’s patient population.

**Table 4:** Barriers to Use, Free Text Survey Data

Domain	DMCDA	SCDA
Knowledge Barriers	-“I don’t have experience using all the meds listed on the decision aid”	-“Don’t know enough about it”
	-“I prefer to send my patients to Endocrinology to discuss treatment options”	-“Often forget it exists”
Attitude Barriers	-“For most of my patients there is an appropriate next step and giving them choices for medications that I would not prescribe doesn’t make sense”	-“Decision aid seems to underestimate benefit”
	-“First choice should be metformin”	-“Patients never choose to use a statin with this decision aid”
	-“A patient may choose a newer pricey medication with the attractive profile with the attitude of ‘Well, I’m not paying for it’”	
External Barrier	-“Computer in rooms often do not pull up the decision aid fast enough”	-“Most patients are already on statins”
	-“Decision aid is slow to load. Laminated cards would be helpful”	
	-“Sometimes discussing options over the phone after test results and hard to use the decision aid remotely”	
	-“I have a very small number of patients on medications for diabetes”	
	-“Most of my patients are already on insulin and/or oral agents”	
	-“Navigating the tool seems a bit clunky”	

Facilitators of decision aid use included clinicians finding them very useful, and their impact on treatment decisions. Both of these facilitators were reported more frequently by clinicians for the SCDA than for the DMCDA (56% vs. 30% and 42% vs 22%, respectively).

For both the SCDA and DMCDA there was some indication that use of the tools as intended for shared decision-making was suboptimal with many clinicians indicating they only discussed the topics they found relevant (60% vs.46%).

## Conclusion

Guidelines for both the treatment of blood cholesterol with a statin and the treatment of hyperglycemia in type 2 diabetes recommend shared decision-making [14, 15]. However, our results indicate that even when decision aids are embedded in the EMR, many clinicians never use these tools. Forty-nine percent of clinicians did not use the SCDA and 91% of clinicians did not use the DMCDA during the 12 months of data collection. Clinician responses to survey questions indicated reasons for lack of use included lack of knowledge of the EMR link, not finding the decision aids helpful, and time constraints. Free text data revealed concerns about lack of alignment of decision aids with guidelines, lack of applicability to patient populations, and in some cases lack of interest of clinicians in involving patients in treatment decisions. These responses point to a lack alignment of values and purpose among clinicians around engaging patients, or a lack of coherence as described by Carl May’s normalization process theory, a conceptual model for how new technologies become embedded in routine practice [16].. Others have also noted lack of coherence as a barrier to successful implementation of shared decision making [17].

Interestingly, our results revealed strikingly different use of the two decision aids by clinicians, with approximately five times more clinicians using the SCDA. This indicates that many clinicians who use a decision aid when deciding on initiation of statin therapy do not do so when deciding on medications for treatment of diabetes. Our survey indicates this may have been at least partially due to lack of knowledge about the existence of the DMCDA. However, the lack of increased use of the DMCDA after the survey by the survey respondents makes it likely that factors intrinsic to the decision aid or to the decision itself are also responsible. Unfortunately, our survey only hinted at what these factors may be. The SCDA was perceived as more useful and as having a larger impact on treatment decisions, but why this is the case is unknown. One potential explanation is that the SCDA automatically calculates the patient’s cardiovascular risk and thus saves the clinicians’ time. However, more clinicians indicated they did not use the SCDA because of time constraints than indicated they did not use the DMCDA due to time pressures. Further research is needed to more fully understand the reasons for differential use.

Importantly, as shown by others [18], our results indicate that use of decision aids does not guarantee shared decision-making. Clinicians indicated they prefer to use the decision aids to discuss only the topics they find relevant, and write-in comments revealed paternalistic views about the desired involvement of patients in treatment decisions. Others have noted poor documentation of shared decision-making when clinicians use links to decision aids from the EMR, perhaps indicating lack of use as intended [19]. Thus even if decision aid use is incentivized through measures such as quality metrics, true patient-centered care will not be achieved without a change in the culture of care. Clinician training in the proper use of decision aids to facilitate shared decision-making, emphasizing the importance of patient engagement in discussions of risk, benefits, alternatives, values and preferences is needed [20]. But perhaps more importance are discussions among clinicians to address divergent views on the value of engaging patients, which would hopefully move clinicians towards coherence in attitudes that promote careful and kind interactions [21].

### Strengths and limitations

The main strength of our study is that it reports on the use of decision aids in routine clinical practice, not in the setting of a trial, making the findings more representative of typical use. The main weakness of the study was that it was done in a tertiary academic practice and thus may lack generalizability to other practice settings.

## Additional file

Additional file 1: Survey Questions (DOCX 18 kb)
