# Supplementary material for: Exploring differences in the use of the statin choice decision aid and diabetes medication choice decision aid in primary care
Source: BMC Med Inform Decis Mak. 2017 Aug 10;17:118. doi: 10.1186/s12911-017-0514-5 (PMC5553736; doi:10.1186/s12911-017-0514-5)
Supplement: Additional file 1: — Survey Questions (DOCX 18 kb) [file 12911_2017_514_MOESM1_ESM.docx]

**SURVEY QUESTIONS**

**Bold text corresponds to Domain and Category in Table 3**

Answers coded as an affirmative response coded as 1

**STATIN CHOICE DECISION AID**

**USE**

**Routine Use**

Do you use the Statin Choice Decision Aid?

- Yes (1)
- No (0)

**BARRIERS**

**Unfamiliar with the Decision Aid**

Before this survey, how familiar or not familiar were you with the Statin Choice Decision Aid, which can be found as a link in GDMS?

- Very familiar (0)
- Somewhat familiar (0)
- Neither familiar nor not familiar (0)
- Somewhat not familiar (1)
- Not at all familiar (1)

Why don’t you use the Statin Choice Decision Aid? (Mark all that apply.)

**Unaware of EMR link**

- I did not know GDMS had a link (1)

**Time Constraints**

- I don't have enough time during a visit to go over this information with my patient. (1)

**Not helpful**

- I am comfortable discussing diabetic treatment options without a decision aid (1)

**Not Accurate**

- I don't feel that the information given in the decision aid is accurate (side effects, pricing, etc.) (1)

**Other reasons for lack of use**

- Other, please specify below: ____________________

**FACILITATORS**

**Useful**How useful or not useful do you find the electronic version of the Statin Choice Decision Aid?

- Very useful (1)
- Somewhat useful (1)
- Neither useful nor not useful (0)
- Somewhat not useful (0)
- Not at all useful (0)

**Appropriate amount of information**

How would you rate the quantity of information presented in the Statin Choice Decision Aid?

- Too much information (0)
- Just the right amount of information (1)
- Too little information (0)

**Often impacts treatment decision**

How often has using the Statin Choice Decision Aid impacted your patient's decision regarding statin treatment?

- Always (1)
- Most of the time (1)
- Sometimes (0)
- Rarely (0)
- Never (0)

**TYPE OF USE**

Which best describes your use of the Statin Choice Decision Aid?

**Only discuss topics patient is interested in**

- I only discuss the topics that my patient is interested in. (1)

**Only discuss topics I find relevant**

- I only discuss the topics that I find relevant for my patient. (1)

**Discuss all topics**

- I go through all the topics with my patient. (1)

**DIABETES MEDICATION CHOICE DECISION AID**

**USE**

**Routinely Used**

Do you use the Diabetes Medication Choice Decision Aid?

- Yes (1)
- No (0)

**BARRIERS**

**Unfamiliar with the Decision Aid**

Before this survey, how familiar or not familiar were you with the Diabetes Medication Choice Decision Aid, which can be found as a link in GDMS? (Unfamiliar with the Decision Aid)

- Very familiar (0)
- Somewhat familiar (0)
- Neither familiar nor not familiar (0)
- Somewhat not familiar (1)
- Not at all familiar (1)

Why don't you use the Diabetes Medication Choice Decision Aid? (Mark all that apply.)

**Unaware of EMR link**

- I did not know GDMS had a link (1)

**Time Constraints**

- I don't have enough time during a visit to go over this information with my patients (1)

**Not helpful**

- I am comfortable discussing diabetic treatment options without a decision aid (1)

**Not Accurate**

- I don't feel that the information given in the decision aid is accurate (side effects, pricing, etc.) (1)

**Other reasons for lack of use**

- Other, please specify below: ____________________

*****

**FACILITATORS**

**Useful**

How useful or not useful do you find the electronic version of the Diabetes Medication Choice Decision Aid?

- Very useful (1)
- Somewhat useful (1)
- Neither useful nor not useful (0)
- Somewhat not useful (0)
- Not at all useful (0)

**Appropriate amount of information**

How would you rate the quantity of information presented in the Diabetes Medication Choice Decision Aid?

- Too much information (0)
- Just the right amount of information (1)
- Too little information (0)

**Often impacts treatment decision**

How often has using the Diabetes Decision Aid Medication Choice impacted your patient's decision regarding diabetes treatment?

- Always (1)
- Most of the time (1)
- Sometimes (0)
- Rarely (0)
- Never (0)

**TYPE OF USE**

Which best describes your use of the Diabetes Medication Decision Aid?

**Only discuss topics patient is interested in**

- I only discuss the topics that my patient is interested in. (1)

**Only discuss topics I find relevant**

- I only discuss the topics that I find relevant for my patient. (1)

**Discuss all topics**

- I go through all the topics with my patient. (1)

**** These questions were included in this section for the Diabetes Medication Choice Decision Aid, but were not included in Table 3 as not also asked for the Statin Choice Decision Aid

- I prefer to send my patients to the Diabetic Educator/Nurse or Endocrinology to discuss treatment options
- I prefer a paper copy (pamphlet or cards) of the decision aid to use in my practice, instead of the electronic version
- I do not manage diabetes
- I do not feel the decision aid is useful
- Other, please specify below: ____________________
